# Androgen receptor (AR) decreases HCC cells migration and invasion via miR-325/ACP5 signaling

**DOI:** 10.7150/jca.49200

**Published:** 2021-01-30

**Authors:** Xiwu Ouyang, Lemeng Feng, Guodong Liu, Lei Yao, Zhiming Wang, Shiqing Liu, Yao Xiao, Gewen Zhang

**Affiliations:** 1 Department of General Surgery, Xiangya Hospital, Central South University, Changsha 410008, China.; 2 Xiangya School of Medicine, Central South University, Changsha, 410013, China.; 3 Department of Geriatric Surgery, Xiangya Hospital, Central South University, Changsha, 410008, China.; 4 National Clinical Research Center for Geriatric Disorders, Xiangya Hospital, Central South University, Changsha, 410008, China.; 5 Department of Respiratory Medicine, Xiangya Hospital, Central South University, Changsha 410008, China.; 6 Key Laboratory of Biological Nanotechnology of National Health Commission, Xiangya Hospital, Central South University, Changsha 410008, China.

**Keywords:** HCC, AR, miR-325, ACP5, migration, invasion

## Abstract

Hepatocellular carcinoma (HCC) is the most 5th commonly diagnosed and 2nd most lethal tumor in the world. The obvious gender advantage of HCC indicates that androgen receptor (AR) may play an important role in the tumor occurrence, develop and metastasis of HCC. Here we found that decreased AR could alter miR-325 to increase ACP5 expression in HCC cells, to increase HCC cells migration and invasion capacities. Mechanism dissection revealed that AR could regulate miR-325 expression through transcriptional regulation and miR-325 might directly target the 3'UTR of ACP5-mRNA to suppress its translation. The *in vivo* orthotopic xenografts mouse model with oemiR-325 also validated *in vitro* data. Together, these findings suggest that AR may decrease HCC progression through miR-325/ACP5 signaling and targeting the AR/miR-325/ACP5 signaling may help in the development of the novel therapies to better suppress the HCC progression.

## Introduction

HCC is the most 5^th^ commonly diagnosed and 2^nd^ most lethal tumor in the world 1. HCC is a malignant tumor with a high recurrence and metastasis rate. It is also a highly lethal tumor, which made it one of the worst cancers in the world. In 2019, it is estimated that there will be 42030 new cases of liver or intrahepatic bile duct tumors and 31780 deaths from HCC in the United States. The incidence rate has increased by two times compared with the past 30 years 2-3. Among them, the incidence of liver cancer in males is more than three times that of females. At the same time, the prognosis of male HCC is usually worse than that of female patients 2-3. For advanced HCC, sorafenib has been proven to be the most effective targeted therapy drug, but its function is regulated by a variety of factors. Therefore, the development of new treatments for HCC is an urgent clinical problem.

The obvious male gender advantage in the onset of HCC indicates that sex hormones and their receptors may play an important role in the tumor occurrence, tumor development and metastasis of HCC 4. The AR signal pathway is complicated in the occurrence and development of HCC 5, and most research results indicate that AR promotes tumorigenesis in the early stage of HCC 6 and inhibits tumor progression in the late stage 7.

MicroRNA is a kind of non-coding RNA with 18-25 nucleotides in length, and it has been proven to induce messenger RNA degradation or inhibit protein translation to negatively regulate the expression of target genes 8, primarily through binding to the 3'UTR of mRNA 9.

Tartrate-resistant acid phosphatase 5 (ACP5), can promote cell movement by regulating focal adhesion kinase phosphorylation, which plays a vital role in bone resorption and osteoclast differentiation. Studies have shown that ACP5 is closely related to the cell invasion, disease progression and distant metastasis of many tumors, such as melanoma and breast cancer 10. However, the related signaling pathways of ACP5 and HCC invasion and metastasis are still unclear.

In this study, we demonstrated that decreased AR could downregulate the expression of miR-325 to promote the migration and invasion capacities of HCC by increasing ACP5 expression, thus we have discovered a new signaling pathway in the AR and HCC progression network, and at the same time, it provides the possibility of a new treatment method for inhibiting the progression of HCC.

## Material and methods

### Cell culture

SK-HEP-1, 293T cell lines were purchased from ATCC. HA22T cell line was gifted from Prof. Yuh-Shan Jou, Academia Sinica, Taiwan. All cell lines were cultured in DMEM (Invitrogen) with 10% Fetal Bovine Serum, 1% penicillin/streptomycin and 1% glutamine and with a condition of a 5% (v/v) CO2 humidified incubator at 37 ºC.

### Lentivirus packaging

The pLKO.1-shAR, pLKO.1-shACP5, pLKO.1-shSMAD3 plasmids were transfected into 293T cells and cultured in the virus room. After 48 hours of transfection, the virus solution was collected and used immediately or frozen in a -80 ºC refrigerator for subsequent use.

The plasmids sequences used were as follows:

- pLKO.1-shAR sequence, Forward: 5'-CCGGGAGCGTGGACTTTCCGGAAATGGATCCATTTCCGGAAAGTCCACGCTCTTTTTG-3'; Reverse: 5'-AATTCAAAAAGAGCGTGGACTTTCCGGAAATGGATCCATTTCCGGAAAGTCCACGCTC-3'.
- pLKO.1-shACP5 sequence, Forward: 5'-CCGGCCTCGGGCAAGTCCCTCTTTAGGATCCTAAAGAGGGACTTGCCCGAGGTTTTTG-3'; Reverse: 5'-AATTCAAAAACCTCGGGCAAGTCCCTCTTTAGGATCCTAAAGAGGGACTTGCCCGAGG-3'.
- pLKO.1-shSMAD3 sequence, Forward: 5'-CCGGGCCTCAGTGACAGCGCTATTTGGATCCAAATAGCGCTGTCACTGAGGCTTTTTG-3'; Reverse: 5'-AATTCAAAAAGCCTCAGTGACAGCGCTATTTGGATCCAAATAGCGCTGTCACTGAGGC-3'.

### RNA extraction and qRT-PCR analysis

Trizol reagent (Invitrogen, Grand Island, NY) was used to extract total RNA, and 2ug of the total RNA was used to reverse transcribe into cDNA by using Superscript III transcriptase (Invitrogen, Grand Island, NY). Relative RNA expression was conducted by Quantitative real-time PCR (qRT-PCR) assay by using a Bio-Rad CFX96 system with SYBR green reagent and the results were normalized by GAPDH.

The primer sequences used were as follows:

- AR primer, Forward: 5'-CCAGGGACCATGTTTTGCC-3'; Reverse: 5'-CGAAGACGACAAGATGGACAA-3'.
- ACP5 primer, Forward: 5'-TGAGGACGTATTCTCTGACCG-3'; Reverse: 5'-CACATTGGTCTGTGGGATCTTG-3'.
- GAPDH primer, Forward: 5'-GGAGCGAGATCCCTCCAAAAT-3'; Reverse: 5'-GGCTGTTGTCATACTTCTCATGG-3'.
- hsa-miR-194-5p primer: TGTAACAGCAACTCCATGTGGA.
- has-miR-512-5p primer: CACTCAGCCTTGAGGGCACTTTC.
- has-miR-6889-3p primer: TCTGTGCCCCTACTTCCCAG.
- has-miR-1227-3p primer: CGTGCCACCCTTTTCCCCAG.
- has-miR-6529-3p primer: CCTGTGCCTTTTACTTCTTTAA.
- has-miR-4266 primer: CTAGGAGGCCTTGGCC.
- has-miR-200a primer: CATCTTACCGGACAGTGCTGGA.
- has-miR-325 primer: CCTAGTAGGTGTCCAGTAAGTGT.
- has-miR-182 primer: TTTGGCAATGGTAGAACTCACACT.
- has-miR-370 primer: CAGGTCACGTCTCTGCAGTTAC.

### Western blot analysis

RIPA lysate was used to lyse the collected cells, and 30 ug protein was electrophoresed in 10% SDS/PAGE gel and then transferred onto PVDF membranes. After that, specific primary antibodies were used to incubate the relative bands for overnight in a 4 ºC room and then incubated with the secondary antibodies for 1 hour and visualized with ECL system (Thermo Fisher Scientific). Alpha-Tubulin (TU-02), GAPDH (6C5), AR (N-20), MMP1 (3B6), MAOA (G-10), SMAD3 (38-Q) and KIF4A (E8) antibodies were purchased from Santa Cruz Biotechnology. RPS4X (ab211427) antibody was purchased from Abcam. ACP5 (DF6989) antibody was purchased from Affinity biosciences. For protein stability assay, SK-HEP-1 cells were transfected with control or AR specific cDNA. After three days, cells were treated with 10 μg/ml of cycloheximide (CHX) for indicated times. ACP5 protein was detected by western blot.

### Cell invasion assay

The invasion experiment is completed with a 24-well plate and an 8 μm chamber system (Corning Life Science). 1×10^5^ cells/well were seeded into upper chamber coated with diluted Matrigel (1:8 dilution, 100 ul/well; BD Biosciences) with serum-free medium and 750 μL media with 10% FBS/well was added into lower chambers for incubation for 36-48 hours. The invaded cells were fixed by methanol and stained by 0.5% (w/v) crystal violet. Each sample was run in triplicate and repeated multiple times.

### Wound-healing migration assay

Cells were seeded into 35-mm plates until they are confluent, and the plates were scraped using a sterile pipette tip to create a wound through the confluent monolayer, then cultured in serum-free medium for SK-HEP-1 with 12 hours and HA22T with 24 hours and photographed at 0 and 12/24 hours, respectively. Measure the distance of wound migration for further analysis.

### Luciferase assay

350 bp fragment of human ACP5 3'UTR with wild or mutant miRNA-responsive elements was cloned into psiCHECK2 vector (Promaga, USA) downstream of the Renilla luciferase ORF. Cells were plated in 24-well plates and transfected the cDNA with Lipofectamine (Invitrogen) as the manufacturer's instruction. Dual-Luciferase Assay (Promega) was used to calculate luciferase activity according to the manufacturer's manual after 48 hours.

### *In vivo* studies

24 6-weeks old nude mouse were divided into 3 groups: 1×10^6^ HA22T plko+vector control (Group 1), HA22T shAR +vector control (Group 2), HA22T shAR+oemiR-325(Group 3), all cells were injected *in situ* under the liver capsule of the left extrahepatic lobe of the mouse. The IVIS system was used to detect tumor growth once a week. The study was carried out under the approval of the ethics committee of Xiangya Hospital Central South University.

### Statistical analysis

All statistical was analyzed by SPSS 22.0 system (SPSS Inc, Chicago, IL). The data values were presented as the mean ± SD. Differences in mean values between two groups were analyzed by two-tailed Student's t test and the mean values of more than two groups were compared with one-way ANOVA. p≤0.05 was considered as statistically significant.

## Results

### AR can decrease the progression of HCC cells

**Figure 1:**
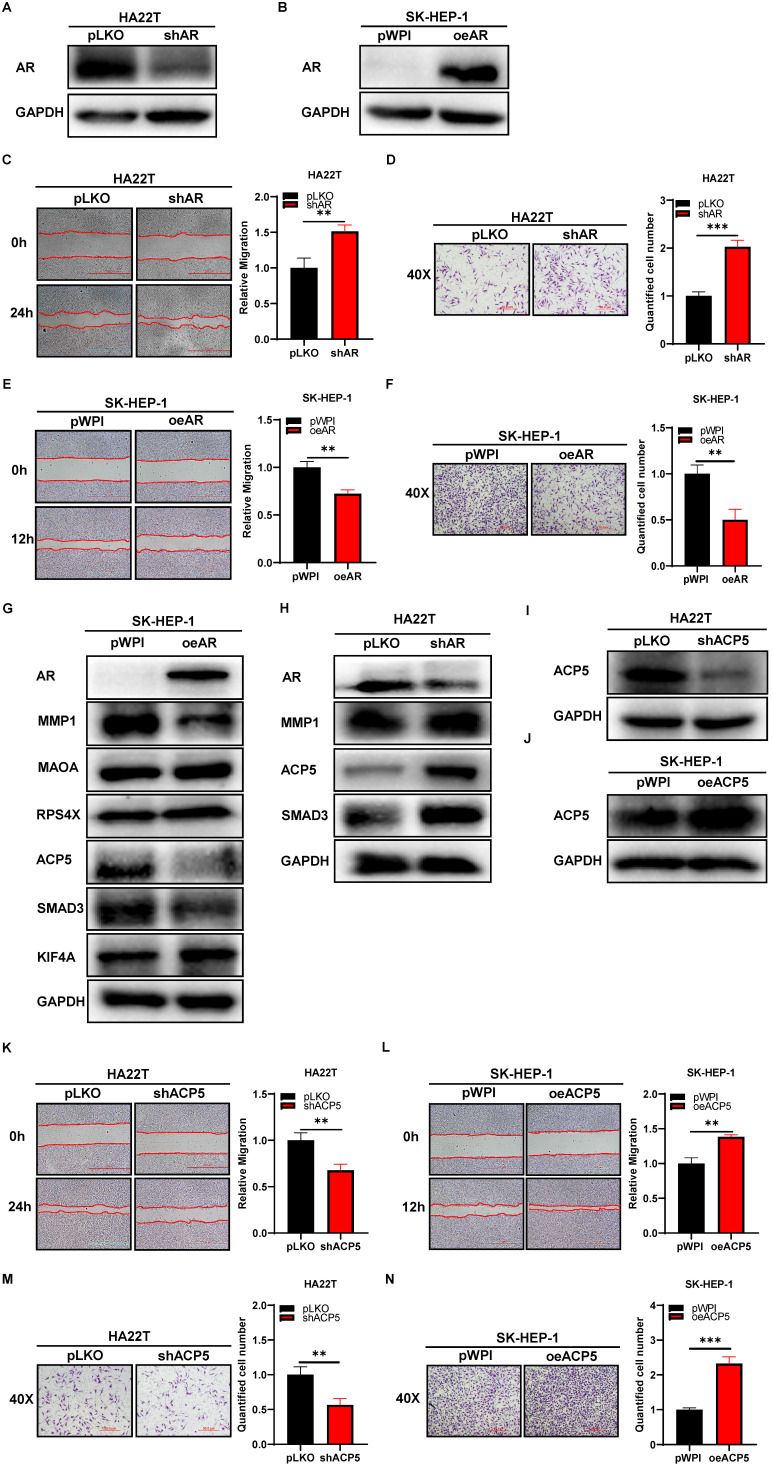
** AR can decrease the progression of HCC cells while ACP5 can increase. A-B.** Western blot was used to check AR protein expression after shAR/oeAR in HCC cells. **C-D.** Wound-healing and transwell invasion assay was used to check the migration and invasion capacity in the HA22T shAR cells. **E-F.** Wound-healing and transwell invasion assay was used to check the migration and invasion capacity in the SK-HEP-1 oeAR cells. **G-H.** Western blot assay was used to check related protein expressions after over expression AR/knocking down AR in HCC cells. **I-J.** Western blot assay was used to check ACP5 expression after shACP5/oeACP5 in HCC cells. **K-L.** Wound-healing assay was used to check the migration capacity after shACP5/oeACP5 in HCC cells. **M-N.** Transwell invasion assay was used to check the invasion capacity after shACP5/oeACP5 in HCC cells. All quantifications are mean ± SD, *p < 0.05, **p < 0.01, ***p < 0.001, ns: no significant difference.

To study the potential effect of AR on HCC cells, we first knocked down AR in HCC HA22T cells (**Figure 1A**) and overexpressed AR in HCC SK-HEP-1 cells (**Figure 1B**), and then we checked AR's effect on HCC progression through wound-healing migration assay and transwell invasion assay. The results showed that knocking down AR could increase HCC cells migration and invasion capacities (**Figure 1C-D**), and overexpressing AR led to decrease HCC cells migration and invasion capacity (**Figure 1E-F**).

Thus, the data from **Figure 1A-F** indicated that AR could decrease HCC cells migration and invasion capacities.

### Mechanism dissection of how AR can decrease HCC cells migration and invasion capacities: via altering the ACP5 expression

**Figure 2:**
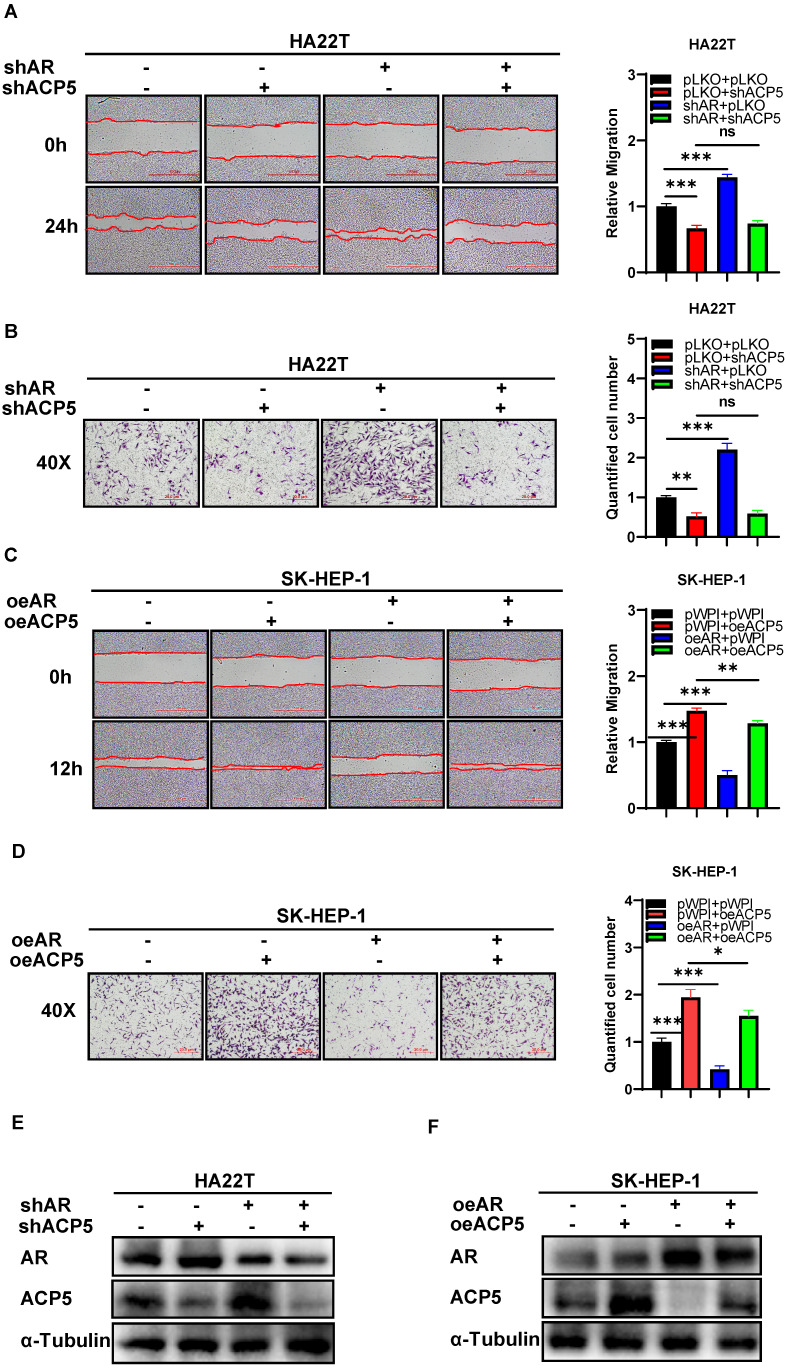
** Mechanism dissection of how AR can decrease HCC cells migration and invasion capacities: via altering the ACP5 expression. A.** Wound-healing assay was used to check the migration capacity after knocking down AR and knocking down ACP5 in HA22T cell.** B.** Transwell invasion assay was used to check the migration capacity after knocking down AR and knocking down ACP5 in HA22T cell. **C-D.** Wound-healing assay and transwell invasion assay were used to check the invasion and migration capacities after oeAR and oeACP5 in SK-HEP-1 cell. **E.** Western blot was used to check AR and ACP5 protein expressions after knocking down AR and knocking down ACP5 in HA22T cell. **F.** Western blot was used to check AR and ACP5 protein expressions after oeAR and oeACP5 in SK-HEP-1 cell. All quantifications are mean ± SD, *p < 0.05, **p < 0.01, ***p < 0.001, ns: no significant difference.

To dissect the mechanism of how AR can decrease HCC cells migration and invasion capacities, we first searched some published papers that were related to cancer progression, and from which, we selected MMP1, MAOA, RPS4X, ACP5, SMAD3 and KIF4A genes, and then we conducted western blot assay to check these genes expressions when knocking down/overexpressing AR in HCC cells. The results showed that ACP5 and SMAD3 might be potential candidate of AR, for their expression was decreased/increased when overexpressing/knocking down AR in HCC cells (**Figure 1G-H**). Then we knocked down/overexpressed ACP5 in HCC cells and checked the migration and invasion capacities in these cells, the results showed that knock down ACP5 could decrease HCC cells migration and invasion capacities and oeACP5 could increase HCC cells migration and invasion capacities (**Figure 1I-N**). Next we conducted reverse assay to checked if shACP5 could really reverse shAR's function to HCC cells, the results showed that shACP5 could partly reverse shAR's function to HCC cells (**Figure 2A-B, E**), and oeACP5 could partly revers AR's function to HCC cells (**Figure 2C-D, F**). For SMAD3, when we shAR/shSMAD3 in HCC cells, the invasion capacity could not be reversed (**Supplementary Figure 1A-B**). Moreover, when we manipulate AR, the mRNA level of ACP5 did not have a consistent change with AR level (**Supplementary Figure 1C**).

Together, the data from **Figure 1G-N** and **Figure 2** indicated that AR can decrease HCC cells migration and invasion capacities: via altering the ACP5 expression.

### Mechanism dissection of how AR can decrease the ACP5 expression in the HCC cells: via altering the miR-325 expression

**Figure 3:**
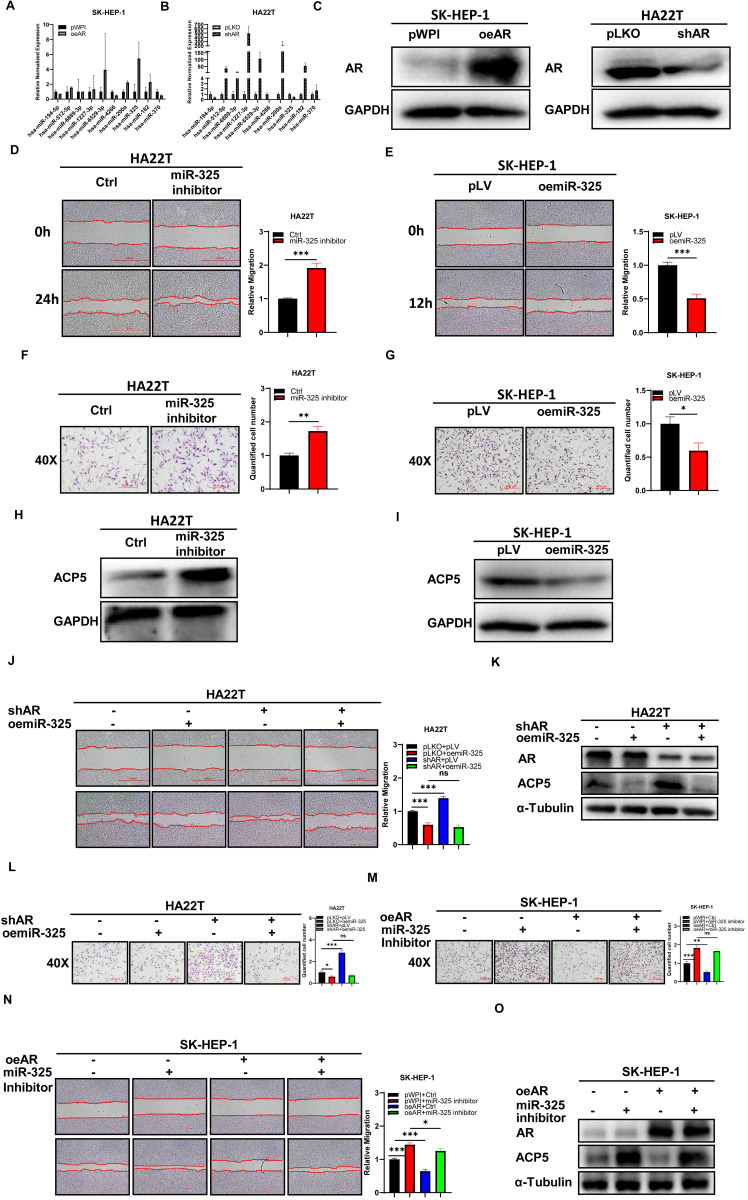
** Mechanism dissection of how AR can decrease the ACP5 expression in the HCC cells: via altering the miR-325 expression. A-B.** qRT-PCR was used to check related miRNAs expression after oeAR/shAR in HCC cells. **C.** Western blot assay was used to check oeAR/shAR efficiency in HCC cells. **D-E.** Wound-healing assay was used to check the migration capacity after adding miR-325 inhibitor/oemiR-325 in HCC cells. **F-G.** Transwell invasion assay was used to check invasion capacity after adding miR-325 inhibitor/oemiR-325 in HCC cells. **H.** Western blot assay was used to check ACP5 expression after adding miR-325 inhibitor in HA22T cells. **I.** Western blot assay was used to check ACP5 expression level after oemiR-325 in SK-HEP-1 cells. **J.** Wound-healing assay was used to check the migration capacity after shAR/oemiR-325 in HA22T cells. **K.** Western blot assay was used to check AR and ACP5 expression after shAR/oemiR-325 in HA22T cells. **L.** Transwell invasion assay was used to check invasion capacity after shAR/oemiR-325 in HA22T cells. **M.** Transwell invasion assay was used to check invasion capacity after oeAR/ adding miR-325 inhibitor in SK-HEP-1 cells. **N.** Wound-healing assay was used to check invasion capacity after oeAR/ adding miR-325 inhibitor in SK-HEP-1 cells. **O.** Western blot assay was used to check AR and ACP5 expression after oeAR/adding miR-325 inhibitor in SK-HEP-1 cells. All quantifications are mean ± SD, *p < 0.05, **p < 0.01, ***p < 0.001, ns: no significant difference.

To dissect the mechanism of how AR can decrease ACP5 expression, we first checked ACP5 stability after overexpressing AR in HCC cells, and the result showed the protein stability was not changed significantly (**Supplementary Figure 1E-F**). Then we focused on miRNAs, for many papers showed that miRNAs that can regulate gene expression by binding to the 3'UTR of specific target mRNAs to inhibit translation or to induce degradation. Firstly, we selected some potential miRNAs that can regulate ACP5 expression through online databases (http://mirdb.org, http://www.microrna.org and http://mirwalk.umm.uni-heidelberg.de/). Then we checked these miRNAs expressions when knock down/overexpressing AR in HCC cells, and the results showed that miR-325 and miR-370 might be the potential candidate to regulate ACP5 expression (**Figure 3A-C**). Next, we added miR-325 inhibitor/overexpressing miR-325 in HCC cells and checked the migration and invasion capacities of them, the results showed that adding miR-325 inhibitor could increase HCC cells migration and invasion capacities and increase ACP5 expression at the same time (**Figure 3D, F, H**), and oemiR-325 could decrease HCC cells migration and invasion capacities and decrease ACP5 expression at the same time (**Figure 3E, G, I**). For miR-370, when we added miR-370 inhibitor into HA22T cell, the ACP5 expression was not increased (**Supplementary Figure 1D**), we suspect it may could not regulate ACP5 expression in HCC cells.

Next, we conducted the reverse assay to check if miR-325 inhibitor/oemiR-325 could reverse oeAR/shAR's function to HCC cells. The results showed that oemiR-325 could partly reverse shAR's function to HCC cells (**Figure 3J-L**), while adding miR-325 inhibitor could partly reverse oeAR's function to HCC cells (**Figure 3M-O**), which indicated that miR-325 could be regulated by AR to regulate HCC progression.

Together, the data from **Figure 3** and**Supplementary Figure 1D-F** indicated that AR could decrease the ACP5 expression in the HCC cells via altering the miR-325 expression.

### Mechanism dissection how AR can alter the miR-325 expression: via transcriptional regulation

**Figure 4:**
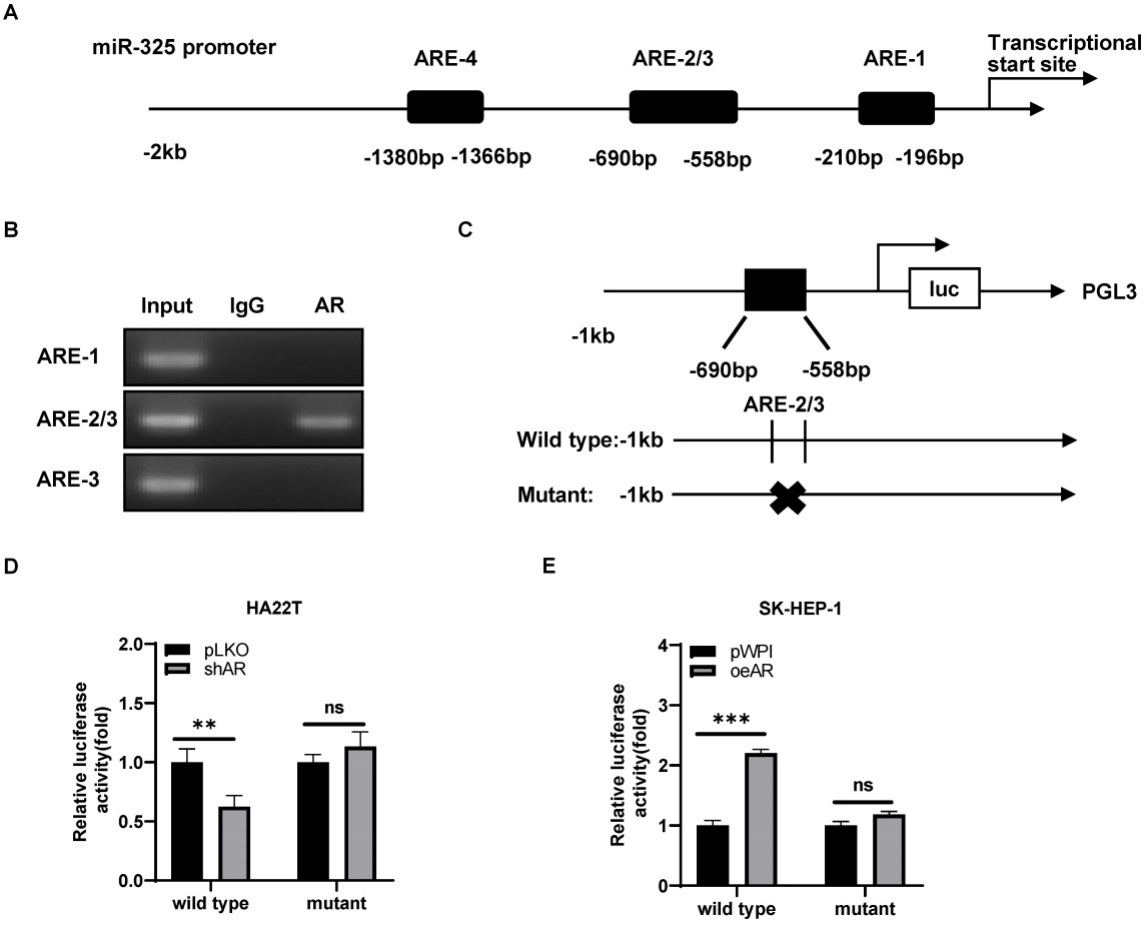
** Mechanism dissection how AR can alter the miR-325 expression: via transcriptional regulation. A.** 4 putative AREs predicted by JASPAR from the miR-325 promoter and ARE motif sequences. **B.** ChIP assay results of 4 AREs of the miR-325 promoter in SK-HEP-1 cells. **C.** The wild type and mutant pGL3-miR-325 promoter reporter constructs. **D-E.** Luciferase activity after transfection of wild type or mutant miR-325 promoter reporter construct in HA22T cells with shAR (D) and SK-HEP-1 cells with oeAR (E) compared to the control cells. All quantifications are mean ± SD, *p < 0.05, **p < 0.01, ***p < 0.001, ns: no significant difference.

To further dissect the molecular mechanism of how AR can regulate the miR-325 expression at the transcriptional level, we searched for the potential AREs on the 2 Kb region of the miR-325 promoter using JASPAR database, and results revealed that 4 putative AREs were located on the regions (-1380~-1366; -690~-676; -572~-558; -210~-196) (**Figure 4A**). We then applied the chromatin immunoprecipitation (ChIP) assay to verify their binding to AR in HCC cells, and results revealed that AR could bind to the ARE-2/3 region (**Figure 4B**).

We then constructed the miR-325 promoter luciferase reporter construct by inserting a 1 kb 5′ promoter region of miR-325 into the pGL3 luciferase backbone as well as a version with the mutated ARE (**Figure 4C**). As expected, the luciferase assay results revealed that knocking down AR significantly decreased luciferase activity in HA22T cells transfected with wild type ARE, but not in the cells with mutant ARE (**Figure 4D**). When we replaced the HA22T cells with the SK-HEP-1 cells, adding AR significantly increased the luciferase activity in the SK-HEP-1 cells transfected with wild type ARE, but not in the cells with mutant ARE (**Figure 4E**).

Together, results from **Figure 4A-E** suggested that AR could increase miR-325 expression at a transcriptional level via binding to the ARE location in its 5′ promoter region of the miRNA precursor.

### Mechanism study of how miR-325 alters the ACP5 expression: via directly target the 3'UTR of ACP5 mRNA

**Figure 5:**
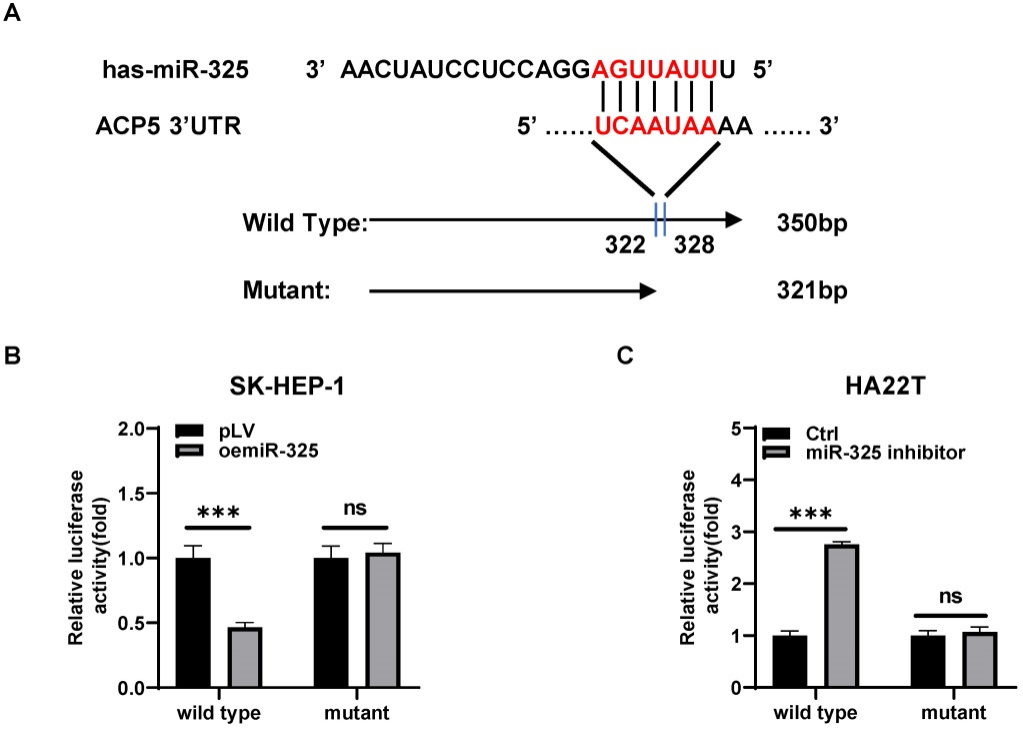
** Mechanism study of how miR-325 alters the ACP5 expression: via directly target the 3'UTR of ACP5 mRNA. A.** Sequence alignment of the ACP5 3'UTR with wild-type versus mutant potential miR-325 targeting sites. **B-C.** Luciferase reporter activity after transfection of wild-type or mutant ACP5 3'UTR reporter construct in SK-HEP-1 and HA22T with oemiR-325 or adding miR-325 inhibitor compared to the control cells. All quantifications are mean ± SD, *p < 0.05, **p < 0.01, ***p < 0.001, ns: no significant difference.

To dissect the mechanism of how AR-miR-325 axis can alter the ACP5 expression at the molecular level, we first searched potential miR-325 targeting site located on the 3'UTR of ACP5-mRNA. We then applied the reporter assay with the psiCHECK2 vector carrying the wide-type 3'UTR and a deletion mutant without the miRNA-target site (**Figure 5A**). Results from the luciferase assay revealed that addition miR-325 inhibitor increased luciferase activity in HA22T cells, while addition of miR-325 decreased luciferase activity in SK-HEP-1 cells transfected with wild type ACP5 3'UTR but not the mutant ACP5 3'UTR (**Figure 5B-C**), suggesting that miR-325 can directly target the 3'UTR of ACP5-mRNA to suppress its protein expression.

### Human clinical study to link the ACP5 to the HCC progression

**Figure 6:**
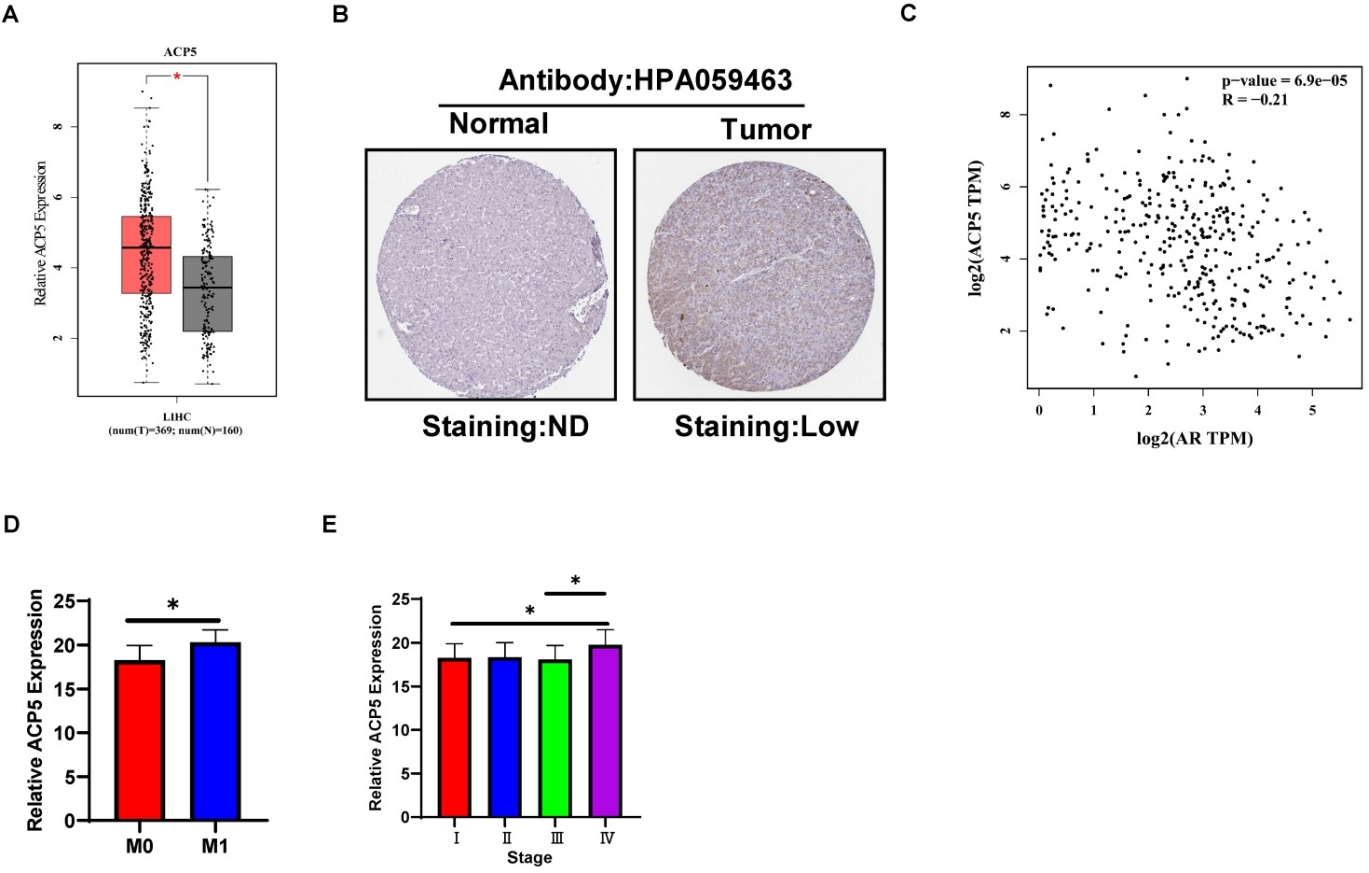
** Human Clinical study to link the ACP5 to the HCC progression. A.** GEPIA website was used to analyze ACP5 expression in normal tissue and primary tumor tissue. **B.** Proteinatlas website was used to analyze IHC data of ACP5 expression in TCGA database. **C.** GEPIA database was used to analyze the correlation between AR and ACP5. **D-E.** ACP5 expression in metastasis and different tumor stages in TCGA database were analyzed. All quantifications are mean ± SD, *p < 0.05, **p < 0.01, ***p < 0.001, ns: no significant difference.

To link the above *in vitro* results with human HCC progression, we used GEPIA website (http://gepia.cancer-pku.cn/) to analyze ACP5 expression in normal liver tissues and HCC samples in TCGA database. The results showed that ACP5 expression was higher in primary tumor samples than in normal samples (**Figure 6A**). Then we used Protein atlas website (https://www.proteinatlas.org/) to analyze IHC data of ACP5 expression in HCC samples, and the IHC data showed that ACP5 expression was higher in primary tumor samples than in normal samples (**Figure 6B**). Next, we used GEPIA website to analyze the correlation between AR and ACP5, the result showed that the expression of ACP5 had negative correlation with AR (**Figure 6C**). Moreover, we analyzed the ACP5 expression in metastasis and different tumor stage in TCGA database, the results showed that ACP5 expression was higher in M1 (with metastasis) tumor than in M0 (no metastasis) tumor (**Figure 6D**), and ACP5 expression was higher in stage IV tumor than in stage I and stage III tumors (**Figure 6E**).

### Preclinical study using *in vivo* mouse model to prove the roles of AR/miR-325/ACP5 axis in the HCC progression

To link our *in vitro* studies to the clinical significance, we first knocked down AR in HA22T cells and got the stable transfected cells, then we transfected the HA22T shAR cells with luciferase reporter gene [for *in vivo* imaging system (IVIS)] to detect tumor progression inside mouse and then transfected the HA22T shAR cells with vector control or oemiR-325 plasmids. The HCC cells were divided into three groups, HA22T plko+vector control (Group 1), HA22T shAR +vector control (Group 2), HA22T shAR+oemiR-325 (Group 3). Tumors growth and metastasis were monitored weekly via IVIS analysis.

**Figure 7:**
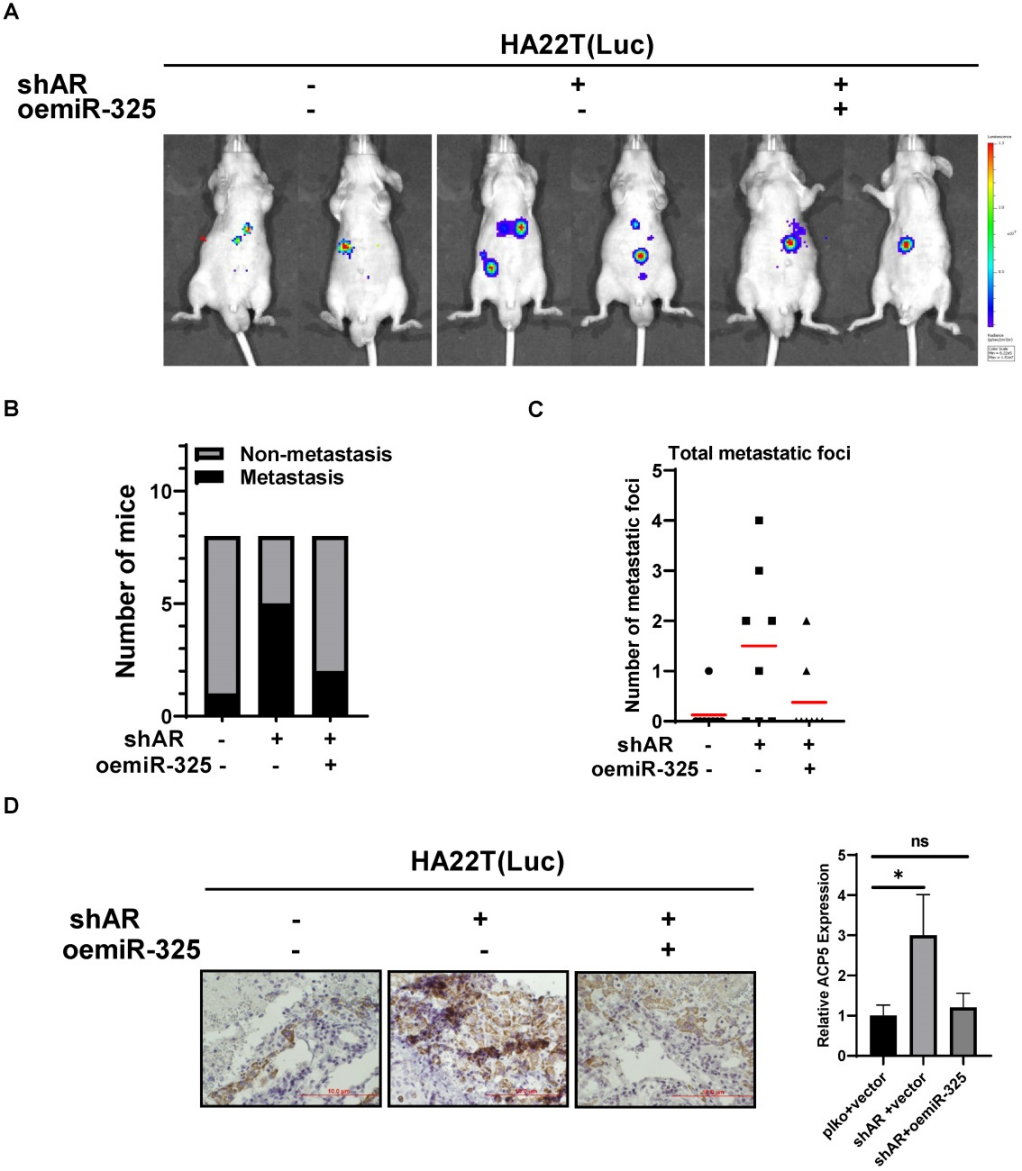
** Preclinical study using *in vivo* mouse model to prove the roles of AR/miR-325/ACP5 axis in the HCC progression. A.** HA22T cells were transduced with Luciferase and with either pLKO/shAR or vector control/ oemiR-325 and then injected into left lobe of liver and IVIS imaging was used to determine the tumor growth and metastasis in mice in each group. **B.** The number of mice with metastasis was counted. **C.** The total number mice with metastatic foci was counted. **D.** IHC staining of ACP5 in different groups. All quantifications are mean ± SD, *p < 0.05, **p < 0.01, ***p < 0.001, ns: no significant difference.

Our results revealed that xenografts of HCC cells transfected with shAR plasmid (Group 2) had more progression than control group (Group 1), and oemiR-325 could partly reverse *in vivo* function of knocking down AR (**Figure 7A**), we also count the mice with metastasis tumors, the results also showed that knocking down AR could promoter tumor metastasis (**Figure 7B**), and we also counted the metastatic foci in each mice, the results showed the same tendency with Figure 7B (**Figure 7C**), what's more, the IHC data of ACP5 expression also confirmed our conclusions (**Figure 7D**).

Taken together the data from **Figure 7** suggests that AR/miR-325/ACP5 axis played a critical role to regulate HCC progression, and targeting this newly identified signaling with oemiR-325 led to suppression of HCC progression.

## Discussion

HCC is one of the most lethal malignant tumors in the world. In 2019, it is estimated that there will be 42030 new cases of liver or intrahepatic bile duct tumors and 31780 deaths from HCC in the United States 3. The high incidence and mortality of HCC makes it one of the most burdened health problems in the world. So far, we still lack effective treatments for advanced HCC. Although Sorafenib is proven to be effective in some patients, but its effect is usually very limited. Therefore, the discovery of new therapeutic targets and the development of new therapeutic drugs are essential for the treatment of liver cancer.

The role of androgen/AR signaling in HCC initiation and progression appears to be contradictory. Studies with genetically modified animals with altered AR expression indicated that AR promoted HBV-, HCV-, and carcinogen-induced HCC initiation 11-13. Most research results indicate that AR promotes tumorigenesis in the early stage of HCC 6 and inhibits tumor progression in the late stage 7, 14. In our study, we found that decreased AR expression could increase the migration and invasion capacities in HCC cells, which was another study to prove AR seems to be a tumor suppressor for HCC in late stage.

MicroRNA is a kind of non-coding RNA with 18-25 nucleotides in length, and it has been proven to induce messenger RNA degradation or inhibit protein translation to negatively regulate the expression of target genes 8, primarily through binding to the 3'UTR of mRNA 9, 15, 16. Deregulated miRNAs are often indicators of cellular event that contribute to the onset of malignancy such as tumor 17. MiR-325 has been reported to be related to the progression of many cancers. Shiiba et al. showed that miR-325 might play a role in head and neck squamous cell carcinoma 18, Tang et al. suggested that the miR-325 may potentially function as a prognostic marker for prostate cancer 19, Wong et al. implied that miR-325 might be involved in HCC malignancy 20, Li et al. found that microRNA-325 could inhibit hepatocellular carcinoma progression by targeting high mobility group box 1 21. In our study, we found that decreased AR could decrease miR-325 expression, and then increase HCC migration and invasion capacities by targeting 3'UTR of ACP5, Moreover, oemiR-325 in HCC cells could partly reverse decreased AR's function both in *in vitro* and *in vivo* experiments, which strongly proved miR-325's negative roles in regulating tumor progression.

ACP5 can promote cell movement by regulating focal adhesion kinase phosphorylation, which plays a vital role in bone resorption and osteoclast differentiation. Scott et al. showed that ACP5 promotes the invasion and distal metastases of melanoma and breast cancer cells 10. Both Chao TY et al. and Wu et al. proved that ACP5 could serve as a marker of bone metastases in breast cancer 22-23. Our study proved that decreased AR could increase HCC cells migration and invasion capacities by up-regulating ACP5 expression, this conclusion was strengthened by the outcome in response to the ACP5-shRNA which could block the biochemical and cell behavior induced by AR in the HCC tumor microenvironment.

In conclusion, AR may decrease HCC progression via altering miR-325/ACP5 signaling, and miR-325 can target the 3'UTR of ACP5-mRNA to suppress its expression. A potential therapy to target this newly identified signaling may help improve the treatment to better suppress the HCC progression.

## Supplementary Material

Supplementary figure S1.
