# Supplementary material for: Androgen receptor (AR) decreases HCC cells migration and invasion via miR-325/ACP5 signaling
Source: J Cancer. 2021 Jan 30;12(7):1915–25. doi: 10.7150/jca.49200 (PMC7974538; doi:10.7150/jca.49200)

Figure S1. A. Transwell invasion assay was used to check invasion capacity after shAR/shSMAD3 in HA22T cells. **B.** Western blot assay was used to check AR and SMAD3 expression after shAR/shSMAD3 in HA22T cells. **C.** qRT-PCR assay was used to check AR and ACP5 expression after shAR/oeAR in HCC cells. **D.** Western blot assay was used to check ACP5 expression after adding miR-370 inhibitor in HA22T cells. **E-F.** Western blot assay was used to check ACP5 protein stability after oeAR in SK-HEP-1 cells. All quantifications are mean ± SD, $*p < 0.05$, $**p < 0.01$, $***p < 0.001$, ns: no significant difference.

Supplementary Figure 1

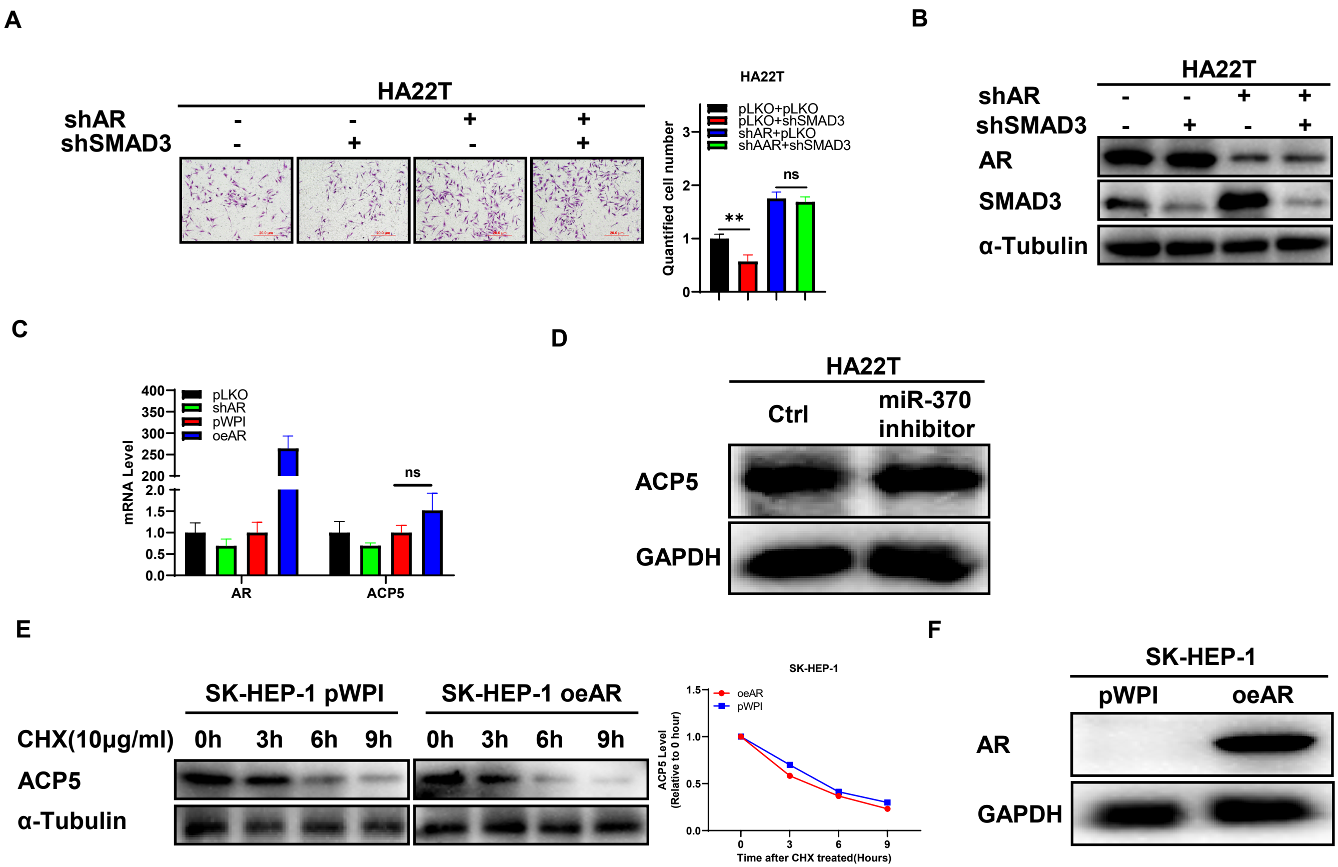

Supplement: Supplementary file 1 — Supplementary figure S1. [file jcav12p1915s1.pdf]
